# A Fast Transform for Brain Connectivity Difference Evaluation

**DOI:** 10.1007/s12021-021-09518-7

**Published:** 2021-04-12

**Authors:** Massimiliano Zanin, Ilinka Ivanoska, Bahar Güntekin, Görsev Yener, Tatjana Loncar-Turukalo, Niksa Jakovljevic, Olivera Sveljo, David Papo

**Affiliations:** 1 Instituto de Física Interdisciplinar y Sistemas Complejos IFISC (CSIC-UIB), Campus UIB, 07122 Palma de Mallorca Spain; 2 Faculty of Computer Science and Engineering, Ss. Cyril and Methodius University, 1000 Skopje, Macedonia; 3 School of Medicine, Department of Biophysics, Istanbul Medipol University, Istanbul, Turkey; 4 REMER, Clinical Electrophysiology, Neuroimaging and Neuromodulation Lab., Istanbul Medipol University, Istanbul, Turkey; 5 Department of Neurology, Dokuz Eylül University Medical School, Izmir, Turkey; 6 Brain Dynamics Multidisciplinary Research Center, Dokuz Eylül University, Izmir, Turkey; 7 Faculty of Medicine, Izmir School of Economics, Izmir, Turkey; 8 Faculty of Technical Sciences, University of Novi Sad, Novi Sad, Serbia; 9 Diagnostic Imaging Center, Oncology Institute of Vojvodina, Sremska Kamenica, Serbia; 10 Fondazione Istituto Italiano di Tecnologia, Ferrara, Italy; 11 Department of Neuroscience and Rehabilitation, Section of Physiology, University of Ferrara, Ferrara, Italy

**Keywords:** Functional brain connectivity, Complex networks, Link difference ranking, Alzheimer’s disease, Schizophrenia

## Abstract

Anatomical and dynamical connectivity are essential to healthy brain function. However, quantifying variations in connectivity across conditions or between patient populations and appraising their functional significance are highly non-trivial tasks. Here we show that link ranking differences induce specific geometries in a convenient auxiliary space that are often easily recognisable at mere eye inspection. Link ranking can also provide fast and reliable criteria for network reconstruction parameters for which no theoretical guideline has been proposed.

## Introduction

Specific anatomical and dynamical connectivity patterns are an essential ingredient of healthy brain functioning (Varela et al. 2001; Braitenberg and Schüz 2013). Conversely, dysconnectivity, i.e. both reduced and increased connectivity, has been suggested to underlie several neurological and psychiatric conditions (Friston 1998; Hahamy et al. 2015; Hillary and Grafman 2017; Hohenfeld et al. 2018). Moreover, the topological properties of the networks induced by anatomical and dynamical connectivity (Bullmore and Sporns 2009) have been shown to be modulated as a function of different stages of development (Cao et al. 2017) and ageing (Meunier et al. 2009), as well as in various neurological and psychiatric pathologies (Fornito et al. 2015).

Quantifying meaningful differences in brain connectivity between given experimental conditions or populations, and determining which network property is important in their identification, are non-trivial tasks, which require either sophisticated statistical testing or computationally intensive machine learning techniques (Zanin et al. 2016) and for which no graphical representation is available. One deep reason for this difficulty relates to the fact that observable dynamical patterns of brain activity emerge in a non-trivial and non-local way from brain connectivity at all scales (Kozma and Freeman 2016). Likewise, while brain topography plays an important role in brain function, topological network properties are essentially statistical in nature. The network neuroscience literature typically emphasises the connectivity and topology induced by strong links. However, weak links have been shown to have a strong impact on network topology, where their inclusion can induce transitions from fractal to small-world universality classes (Gallos et al. 2012), but also on the dynamics of and processes taking place on the network (Csermely 2004; Karsai et al. 2014). Taken together, these considerations suggest that experimental conditions may be identifiable not just through the structure induced by strong links, with the possible addition of weak ones, but via their relations across the whole network in a way that is at least partially independent of topographical localisation.

Here, we propose a computationally feasible method to quantify differences in connectivity across experimental conditions based on link weight rankings. The links of equal-sized all-to-all weighted dynamical networks of brain activity associated with two different experimental conditions are ranked according to their weight. The median and standard deviation of these links are then compared across conditions. Link ranking differences at all levels of the rank-weight distribution turn out to induce specific geometries in a convenient auxiliary space, whose axes are respectively their between-group difference in median and standard deviation. Ultimately, the data are represented in a scatter plot, where each point corresponds to a link ranking, and its position to the difference in the link median / standard deviation between groups. This graphical representation allows to easily depict how connectivity strengths are modified by a condition, both in terms of median value and variability; whether such changes are uniform, or centred on strong / weak links; and how certain these results are.

## Methods

### Creating the Representation

**Fig. 1:**
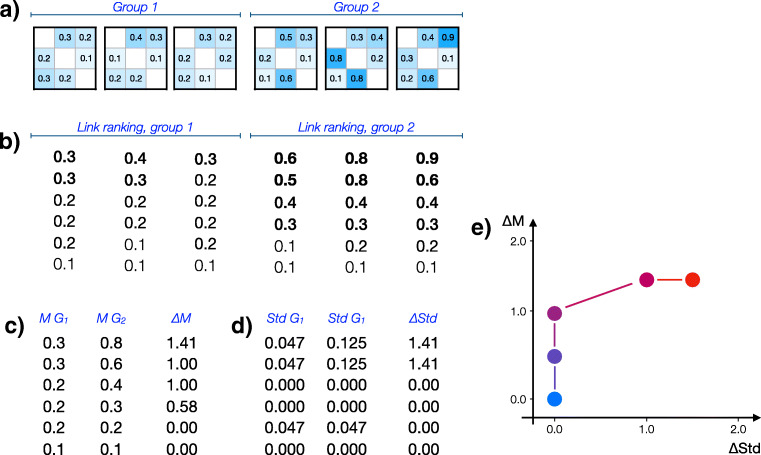
Graphical representation of the proposed method. **a** Initial connectivity matrices, for two groups composed of three networks each. **b** Ranking of each network’s links. **c** Median of the link’s values, for each group and each ranking position; and difference of link’s medians, calculated as the logarithm in base two of the ratio between the values of both groups. **d** Standard deviation of the link’s values, for each group and each ranking position; and difference of link’s standard deviations. **e** Final graphical representation in the Δ*M* −Δ*Std* plane, each point representing a ranking position, and coloured according to the ranking position (from red, highest, to blue, lowest)

The method takes as input a set of weighted adjacency matrices, each representing the connectivity between different brain regions of a given subject. Note that no restrictions are imposed on the way such connectivity is calculated, provided the result takes the form of a real number. Two different groups of subjects (e.g. patients and controls) are compared, with each matrix belonging to one of them - see Fig. 1a. Note that the values in the adjacency matrices can be obtained through any connectivity metric (including, for instance, linear correlation or Granger causality (Bressler and Seth 2011)), provided the previous condition is fulfilled, i.e. that the metric yields a real scalar value for each pair of nodes. Also, note that Fig. 1a presents a very simple case, with three networks in each group, e.g. reconstructed from neuroimaging recordings of three trials of healthy patients and three trials of patients. Link weights (without self-links) are then extracted from each matrix, and ranked in decreasing order - Fig. 1b. For each group and ranking position, two metrics are further extracted: the median (Fig. 1c) and the standard deviation (Fig. 1d) of link weights within the same group.

The difference in the two metrics between two groups is respectively calculated as ${\Delta } M = \log _{2} M_{g1} / M_{g2}$ (Fig. 1c, third column) and ${\Delta } Std = \log _{2} Std_{g1} / Std_{g2}$ (Fig. 1d, third column), with *M* representing the median, and *g*1 and *g*2 indicating the two groups to be compared. In both cases, values of Δ greater (respectively, smaller) than zero indicate that networks in the second group have larger (smaller) values than those in the first. Note that the values of Δ*M* and Δ*Std* are still dependent on the ranking position. Finally, all results are represented in a scatter plot, where points, corresponding to ranking positions, are located in a Δ*Std* - Δ*M* plane (Fig. 1e). For the sake of clarity, points are coloured according to their position in the ranking, from blue (weakest links) to red (strongest links).

**Fig. 2:**
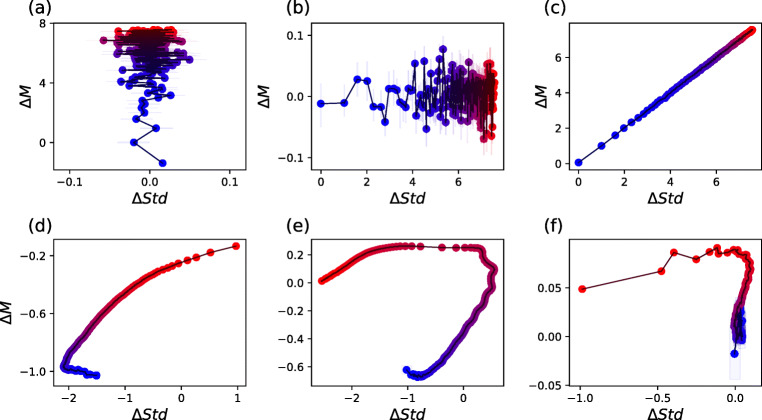
Analysis of the results yielded by a set of six synthetic models. In the first three (i.e. the *static* ones, top row) link weights are directly assigned, while in the last three (i.e. the *dynamic* ones, bottom row) they are derived from the corresponding nodes’ time series

The final picture, as the one in Fig. 1e, can be interpreted as follows. Points form a continuum ranging from strongest (red) to weakest (blue) links, and their position indicates how weights differ between conditions. Positive values along the Y axis indicate that the second group has stronger links on average; and positive values along the X axis, that the second group has a larger variability. As an additional example, consider the first panel of Fig. 2 (i.e. Model 1). Here, stronger links (i.e. red points) in the first group have the same median link strength (as Δ*M* ≈ 0) and higher variability (Δ*Std* ≈ 1) than links in the second group. On the other hand, weak links (i.e. blue points) have both a lower median strength (Δ*M* ≈− 1) and lower variability (Δ*Std* ≈− 2).

For the sake of completeness, an additional feature is added to the scatter plot. A semi-transparent box is plotted alongside each point, spanning from the 16th to the 84th percentile of each metric calculated by taking half of the available matrices at random. Thus, each box represents the uncertainty in the position of the corresponding point if only half of the data were available.

### Data Sets Description

The nine data sets considered in this study are described in what follows - a review of their main characteristics is reported in Table 1. They have been selected to cover the main neuroimaging techniques whose outputs are customarily interpreted as networks, and thus provide a wide set of use cases. Still, each data set has been analysed independently of the others. Unless otherwise specified, no further processing steps (including noise reduction or artefact elimination) have been performed.

**Table 1:** Main characteristics of the considered data sets

Type	Data set	# controls	# patients	# sensors / ROIs	Length
EEG	Schizophrenia	14	14	19	≈ 15m
	Alzheimer’s disease	38	42	32	≈ 8m
	Parkinson’s disease	22	74	32	≈ 8m
	Alcoholic	4,024	7,033	64	1s
fMRI	Autism	592	521	116	5-16 m
	Schizophrenia	33	21	116	6m
	Alzheimer’s disease	34	21	116	6m
DTI	Autism	43	51	264	*n.a.*
	Gender	114	80	188	*n.a.*

*# controls* and *# patients* respectively refer to the number of control subjects and patients available for this study, except in the case of the Alcoholic EEG data set, for which they represent the number of trials

#### Schizophrenia (EEG)

This data set includes resting state EEG recordings from a set of schizophrenia patients and matched control subjects, as described in Ref. Olejarczyk and Jernajczyk (2017) and available at 10.18150/repod.0107441. The 14 patients (7 males, 27.9 ± 3.3 years, and 7 females, 28.3 ± 4.1 years) met International Classification of Diseases ICD-10 criteria for paranoid schizophrenia (category F20.0). The 14 corresponding healthy controls were 7 males, age of 26.8 ± 2.9 years, and 7 females, age of 28.7 ± 3.4. Fifteen minutes of EEG data were recorded during an eyes-closed resting state condition. Data were acquired at 250Hz using the standard 10-20 EEG montage with 19 EEG channels: Fp1, Fp2, F7, F3, Fz, F4, F8, T3, C3, Cz, C4, T4, T5, P3, Pz, P4, T6, O1, O2. The reference electrode was placed at FCz.

#### Alzheimer’s Disease (EEG)

The EEG data set of Alzheimer’s disease (AD) patients was recorded at Istanbul Medipol University Hospital in Istanbul and the Izmir Dokuz Eylul University Multidisciplinary Brain Dynamics Research Center in Izmir. AD patients were diagnosed according to the criteria of the “National Institute of Neurological and Communicative Diseases and Stroke-Alzheimer’s Disease and Related Disorders Association (NINCDS-ADRDA)” (McKhann et al. 1984; McKhann et al. 2011) and the “Diagnostic and Statistical Manual of Mental Disorders-4th Edition (DSM-IV-TR)” (APA et al. 2013). Also, the Clinical Dementia Rating (CDR) scale (Berg 1988; Gurvit and Baran 2007; Morris 1993; 1997) was used for assessing the severity of AD. A total of 42 patients (ages: 56 − 86, median of 74; gender: 6 male; edu: 0 − 13, median of 5) and 38 healthy control subjects (ages: 54 − 70, median of 62.5; gender: 5 male; edu: 0 − 12, median of 5) have here been analysed. The Mini-Mental State Examination (MMSE) test (Folstein et al. 1983; Gungen et al. 2002) was used to evaluate the general cognitive state of all participants. AD-related medicine use was not intervented in the patient group and the patients with AD were taking cholinergic monotherapy or combined cholinergic treatment with memantine.

The EEG of all healthy controls and AD patients were recorded in a dimly isolated room with a Brain Amp 32-channel DC system machine (Brain Product GmbH, Germany) from 32 different electrodes which were arranged according to the international 10/20 system. The sampling rate was 500 Hz with band limits of 0.01 - 250 Hz. All impedances were kept below 10 Kohm and two additional linked earlobe electrodes (A1+A2) served as reference electrodes. Electro-ocologram was recorded with two electrodes placed in the medial upper and lateral orbital rim of the left eye.

#### Parkinson’s Disease (EEG)

The EEG data set of Parkinson’s patients was recorded at Istanbul Medipol University Hospital in Istanbul. PD patients were diagnosed according to the criteria of “United Kingdom Parkinson’s Disease Society Brain Bank” (Daniel and Lees 1993). The Unified Parkinson’s Disease Rating Scale (UPDRS) (Lang and Fahn 1989) was used in order to determine the clinical features of PD; and the Hoehn-Yahr scale (Hoehn and Yahr 1967) was used to determine the disease stage. A total of 74 patients (ages 56 − 86, median of 74) and 22 matched control subjects (ages 54 − 89, median of 67) have here been analysed. All patients with PD were evaluated 60 to 90 minutes after their morning dose of levodopa for the EEG recordings. Recording conditions, equipment and electrodes location are as in the Alzheimer’s disease (EEG) data set.

#### Alcoholic (EEG)

This data set contains EEG recordings from a group of alcoholic subjects and matched controls (Zhang et al. 1995; Cao et al. 2014), freely available at https://archive.ics.uci.edu/ml/datasets/EEG+Database. Each trial corresponds to an object recognition task, as described in Snodgrass and Vanderwart (1980); and its corresponding EEG activity has been recorded during one second, with a 256 Hz (3.9-ms/epoch) sampling rate from 64 electrodes located at standard scalp sites. 4,024 trials for controls and 7,033 for patients are available, for a total of 11,057 instances. Note that trials are here assumed to be independent from each other, even when coming from the same subject; therefore, and for the purpose of this study, the 11,057 instances are equivalent to recordings coming from 11,057 different subjects.

#### Autism (fMRI)

The first fMRI dataset selected for this study is ABIDE II (Di Martino et al. 2017), available at http://fcon_1000. projects.nitrc.org/indi/abide/abide_II.html, which consists of a collection of 19 datasets of individuals with Autism Spectrum Disorder (ASD) and typical controls (TC). It includes resting state functional magnetic resonance imaging (rs-fMRI) data, diffusion tensor imaging (DTI), phenotypic data, in addition to anatomical data. To create functional connectomes representations we have taken anatomical and functional data from all 19 datasets (except longitudinal collections), for 521 ASD patients and 592 TC and a grand total of 1113 subjects. Additional information about these subjects and their corresponding image acquisitions is given at http://fcon_1000.projects.nitrc.org/indi/abide/abide_II.html.

The preproccessing was performed using Statistical Parametric Mapping (SPM12) (Neuroimaging 2016) in MATLAB 2018b incorporated into the CONN toolbox (Whitfield-Gabrieli and Nieto-Castanon 2012). The preprocessing steps include a default pipeline with functional realignment (motion estimation and correction), slice-timing correction, coregistration to subjects respective anatomical (T1) images with normalisation to the standard Montreal Neurological Institute (MNI) template, outlier detection, and smoothing with an 8 mm full width at half maximum (FWHM) kernel. In addition to these steps, segmentation of grey matter, white matter, and cerebrospinal fluid (CSF) areas was employed for the removal of temporal confounding factors (white matter and CSF). Moreover, band-pass filtering was performed with a frequency window of 0.008-0.09 Hz. For head motion and artefacts elimination, outlier time points were identified in the motion parameters and global signal intensity using ART (Whitfield-Gabrieli et al. 2011) added to the default pipeline in CONN toolbox. ROI-to-ROI 116x116 functional connectivity matrices for each subject were calculated using CONN toolbox with the use of Anatomical Atlas Labeling (AAL) template (Tzourio-Mazoyer et al. 2002) for brain atlas ROI parcellation.

#### Schizophrenia (fMRI)

The second fMRI dataset included in this study is the open source COBRE data set (http://fcon_1000.projects.nitrc. org/indi/retro/cobre.html), consisting of anatomical and resting state functional magnetic imaging (rs-fMRI) data for 72 Schizophrenia patients (SZ) and 75 typical controls (TC). The rs-fMRI data were obtained using single-shot full k-space echo-planar imaging (EPI) with TR= 2 s, TE= 29 ms, matrix size= 64x64, slice number= 32 slices, and voxel size= 3x3x4 mm3. Additional information on the dataset images acquisitions and phenotypical data is available at http://fcon_1000.projects.nitrc.org/indi/retro/cobre.html. The executed preprocessing coincide with the one of the Autism (fMRI) data set.

#### Alzheimer’s Disease (fMRI)

The Alzheimer’s Disease Neuroimaging Initiative (ADNI) (www.adni-info.org) was launched in 2003 with a primary goal to test whether serial magnetic resonance imaging (MRI), positron emission tomography (PET), biological markers, and clinical and neuropsychological assessment can be combined to measure the progression of mild cognitive impairment (MCI) and early Alzheimer’s disease (AD).

In this study we used functional magnetic resonance (fMRI) datasets from the ADNI database (http://adni. loni.ucla.edu). The 60 recordings used in this study were from the control (pre-treatment) sessions. The participants were categorised according to the clinical data in multiple groups, out of which in this study two groups were used: cognitively normal subjects’ (CN) group (37 participants), and Alzheimer’s dementia (AD) group (23 participants). The CN group comprises 17 males and 20 females of an average age 73.65 ± 5.74, whereas the AD group comprises 10 males and 13 females of an average age 73.17 ± 7.62. Each participant was scanned on a 3.0T Philips MRI Scanner. fMRI axial images were obtained using echo planar (EPI) sequence with repetition time (TR) of 3000ms; echo time TE = 30ms; flip angle (FA)= 800, and 48 slices with slice thickness of 3.313 mm. Participants were instructed to relax and keep eyes closed during the scanning session. A total of 140 volume data were available for each participant.

The dataset was preprocessed using the Data Preprocessing Assistant for Resting-State fMRI (DPARSF) software (http://www.rfmri.org/DPARSF) (Chao-Gan and Yu-Feng 2010). The first seven time points were discarded to ensure the stabilisation of the magnetic field. Preprocessing steps include: slice timing correction, realignment to eliminate movement artefacts and spatial normalisation to the standard EPI template. Images were spatially smoothed with Gaussian kernel with full width at half-maximum (FWHM) of 6 mm. Both linear and quadratic trends were removed and head motion parameters, cerebrospinal fluid and white matter signals were regressed out from the data. Participants with significant motion artefacts were excluded from the study, reducing the set to a total of 34 CN and 21 AD participants. Finally, BOLD signals were extracted from 116 brain region according to automated anatomical labelling (AAL) (Tzourio-Mazoyer et al. 2002) atlas.

#### Autism (DTI)

The first diffusion tensor imaging (DTI) dataset with structural connectomes for this study is the UMC database UCLA Autism collection (Rudie et al. 2013) taken from the UCLA multimodal connectivity database (Brown et al. 2012), openly available at http://umcd.humanconnectomeproject.org/. The collection consists of pre-constructed structural and functional connectomes for a total of 175 subjects, out of which 94 are structural connectomes for 51 Autism Spectrum Disorder (ASD) patients and 43 Typically Developing (TD) controls, in addition to 79 functional connectomes for 42 (ASD) patients and 37 TD controls. In this study, we have selected the structural DTI fiber connectivity connectomes 264x264 matrices, for the discovery of differences between ASD and TD. Phenotypical and demographic dataset information, as well as images acquisition and structural DTI fibre connectivity matrices construction details are given in Rudie et al. (2013).

#### Gender (DTI)

The last dataset for this study was also obtained from the UCLA multimodal connectivity database (Brown et al. 2012) (openly available at http://umcd.humanconnectomeproject.org/), and specifically from the NKI Rockland collection. It consists of 194 structural and functional connectivity matrices for 194 control subjects taken from the Nathan Kline Institute (NKI)/Rockland sample dataset (Nooner et al. 2012). Data information, DTI and fMRI preprocessing, as well as structural and functional connectivity matrix derivation are described in more detail in Brown et al. (2012). For the purpose of discovering gender differences (male vs. female) we have only used the structural 188x188 connectomes matrices.

### Functional Network Reconstruction

Once a set of time series has been obtained for each subject, in the case of EEG and fMRI data, these have been divided in non-overlapping windows of size *τ*. Unless otherwise specified, we have here considered *τ* = 128. Finally, a weighted adjacency matrix *W* has been reconstructed for each window, where each element *w*_*i*,*j*_ represents the strength of the functional connectivity between nodes (electrodes or ROIs) *i* and *j*. As previously discussed, any method can be used to estimate such connectivity, provided the output is a scalar number for each pair of nodes. To illustrate how different methods can yield different results, we here consider four metrics commonly used in neuroscience:

- Linear Pearson’s correlation, corresponding to the absolute value of the classical linear correlation between the two time series.
- Granger Causality (GC), a linear causality metric based on evaluating the improvement in the forecast of the time series *Y* when information about a second time series *X* is included. If the error in the prediction is reduced, *X* is said to *Granger-cause* *Y* (Bressler and Seth 2011). The value of each element *w*_*i*,*j*_ is defined as the $-\log _{10}$ of the *p*-value of the Granger test between time series *i* and *j*.
- Mutual Information (MI), a measure of the mutual dependence between the two variables. It is defined as the amount of information obtained about one time series through observing a second one (Kraskov et al. 2004).
- Transfer Entropy (TE), a metric measuring the amount of directed transfer of information between two time series. More specifically, it is defined as how much the uncertainty in future values of a time series *Y* is reduced by knowing the past of a second time series *X* (Vicente et al. 2011).

## Results

### Synthetic Models

As a first test case, and in order to better illustrate the behaviour of the proposed methodology, we here show the results obtained through six synthetic models. These have the advantage of being clearly defined, such that the validity, consistency and meaning of results can easily be checked.

The first three, called *static* in Fig. 2, are based on creating a set of networks with pre-defined link weights - as opposed to be derived from time series. Specifically, each model comprises two groups of 10,000 networks each, each one composed of 20 nodes. An increasing index *l* = 1,2,… is then associated to each link, and the corresponding weight is defined as:

*Panel (a), Static model 1.* The weight of link *l* is drawn from a normal distribution $\mathcal {N}(l, 1)$ for networks in the first group, and $\mathcal {N}(l^{2}, 1)$ for networks in the second one. This makes links in the second network stronger, on average, in a supralinear way - this reflects in high values of Δ*M* for strong links (represented as red points), and in a curve oriented from bottom to top.

*Panel (b), Static model 2.* The weight of link *l* is here drawn from a normal distribution $\mathcal {N}(l, 1)$ for networks in the first group, and $\mathcal {N}(l, l)$ for networks in the second one. Compared to the previous case, the average link strength is kept proportional to *l*, but the corresponding variability is increased in the second group. As should be expected, this results in high values of Δ*Std* for strong links, and a global curve evolving from left to right in the plane.

*Panel (c), Static model 3.* This model combines both previous models, such that link weights are drawn from a normal distribution $\mathcal {N}(l, 1)$ for networks in the first group, and $\mathcal {N}(l^{2}, l)$ for networks in the second one. Accordingly, the result is a diagonal curve, going from small to large values of both Δ*M* and Δ*Std*.

We then move to the analysis of *dynamic* models, i.e. models in which the weight of the link connecting two nodes is derived from the correlation between the time series describing the corresponding nodes’ dynamic. This is more similar to the typical analysis in neuroscience, and further yields more complex results in the Δ*Std*-Δ*M* plane. For that, we again consider the case of two groups of 10,000 networks and 20 nodes, and with a time series of length *τ* = 8 associated to each node. Link weights between pairs of nodes are calculated as the absolute value of the linear correlation between the corresponding time series. In the case of the first group, these time series are always created by mixing a common fixed pattern with a random component. In the case of the node *i*, its time series is given by $x^{(i)}_{t} = \pi _{t} + \alpha ^{(i)} \mathcal {U}(-1, 1)$. *π* is a fixed time series of *τ* = 8 values, created by drawing random numbers from an uniform distribution (0,1), upon which a random signal is superimposed. The amplitude of such additive signal is controlled by *α*^(*i*)^, a random number drawn from a uniform distribution $\mathcal {U}(0, 1)$. Note that each pair of nodes with small *α* will have a similar dynamics, due to the dominance of *π*, and hence a large correlation. Networks of the second group are constructed according to different generative models, with time series defined by $y^{(i)}_{t} = \pi _{t} + {\upbeta }^{(i)} \mathcal {U}(-1, 1)$.

*Panel (d), Dynamic model 1.* β^(*i*)^ is a number drawn from a uniform distribution $\mathcal {U}(0, 8)$. As β is larger than *α*, the noisy component is stronger in the second group, and thus links have a smaller weight - see the negative value of Δ*M*. Globally, weak links tend to have a more negative Δ*M*, as differences are magnified, in relative terms, by the smaller weight; and a smaller variability, as the larger value of β reduces the probability of having a strong link at low ranking positions.

*Panel (e), Dynamic model 2.* β^(*i*)^ is a random number drawn from a uniform distribution $\mathcal {U}(0, 0.2)$ for *i* ≤ 10; and from an exponential distribution with a scale parameter 1/*λ* = 4 otherwise. The first ten nodes are thus characterised by a highly synchronised dynamics and homogeneous weights, thus leading to a positive Δ*M* and negative Δ*Std*; the synchronisation is then lost for the remaining links, recovering a curve similar to that of Model 1.

*Panel (f), Dynamic model 3.* In this case, β^(*i*)^ is a number drawn from a uniform distribution $\mathcal {U}(0, 0.3i)$, and is thus node-dependent: some nodes (small *i*) have a necessarily highly correlated dynamics, while others (*i* close to 20) are more heterogeneous. While the global connectivity strength is mostly constant (i.e. Δ*M* ≈ 0), some pairs of nodes are forced to be synchronised, and hence their variability is reduced (note the negative Δ*Std* for the five strongest links).

### Analysis of Brain Data

**Fig. 3:**
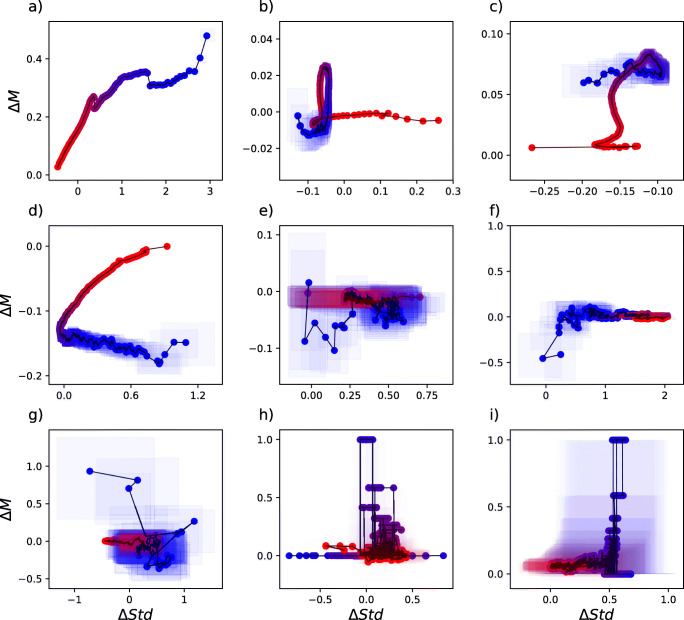
Graphical representations yielded by the proposed methodology for the nine functional and structural brain data sets here considered. Refer to Methods for details on data and processing

We further applied the proposed method to a large collection of anatomical and functional brain data recorded with various neuroimaging techniques from people suffering from a number of neurological and psychiatric pathologies as well as from matched control subjects - see Fig. 3.

Several points are worth mentioning. First, in most cases, the transform yields a non-trivial picture of the overall structure of the differences between a given pathology and its corresponding control group in terms of differences in connectivity strength ranking. This result indicates that, even in the absence of topographic localization information, often deemed necessary in system-level neuroscience, the transform retains enough information of the network connectivity pattern to discriminate pathological signals from healthy control ones.

Second, our results suggest that the relevant discriminant information is encoded in the hierarchy of brain connectivity strength but also, importantly, in its variability. While variability it often paid little attention in network analysis, our results are consistent with its prominent role in healthy biological systems and in their pathology (Goldberger et al. 2002; West 2010).

Third, the geometry of the rank-difference transform in the median-std space presents marked pathology-specificity which can often be appreciated at a glance. For instance, schizophrenia is associated with a substantial increase in both median weight and variability for weak links - note that a Δ*Std* of 3 is equivalent to a eight-fold increase in variability - while the strongest links do not significantly differ from those of control subjects (Fig. 3 panel (a)). The opposite pattern is observed for Alzheimer’s disease (AD) (panel (b)), in which the increase in variability is limited to strong links. Somehow in between the two previous patterns is that of alcoholic patients, with higher variability in the strongest and weakest links, but not in intermediate ones (panel (d)).

Panels (h) and (i) show how anatomical (as opposed to functional) connectivity networks, here obtained through Diffusion Tensor Imaging (DTI), can also be analysed through the proposed methodology. Weak links are the ones showing a major difference in both cases, while strongest links are mostly stable at Δ*M* ≈ 0. Importantly, insofar as most current network reconstruction studies typically filter out weak links, typically retaining only a low percentage of the strongest ones, the results for both functional and anatomical data indirectly suggest that the detection ability of these studies may also be pathology-specific.

**Fig. 4:**
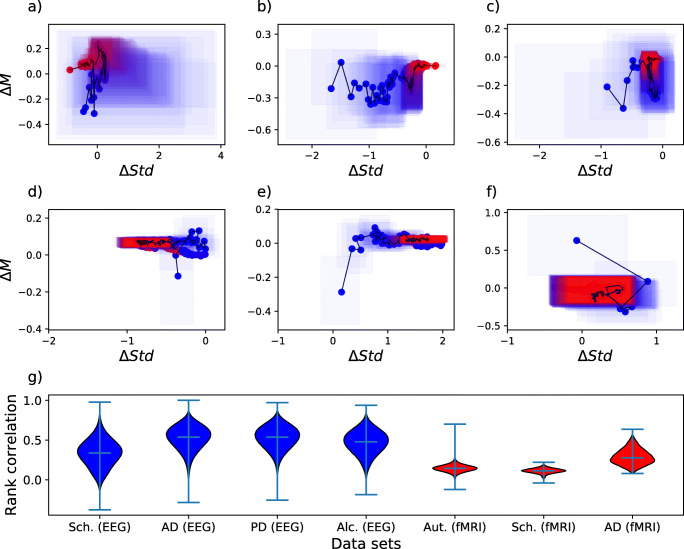
Analysis of the resolution of fMRI data. Panels (**a**), (**b**) and (**c**) report the graphical ranking representations for respectively the Schizophrenia, AD and PD EEG data sets, when the corresponding time series are downsampled by taking one value every 128 - thus simulating a time resolution similar of that of fMRI. The results are fuzzy structures, similar to the ones obtained for the fMRI data sets. Panels (**d**), (**e**) and (**f**) report the ranking representations for the three considered fMRI data sets, when the values obtained are averaged over 4 consecutive links in the ranking. The obtained graphs suggest that the unclear structure is not due to the high number of nodes (i.e. to a too high spatial resolution), but instead to the small time resolution of fMRI time series. Panel (**g**) finally represents violin plots of the distribution of the Spearman rank correlation between the link weight of all control subjects in each data set. In general fMRI rankings (red violins) are less stable than EEG ones (blue violins), contributing to the appearance of fuzzy structures

Finally, the results appear to be consistently more clear-cut for electroencephalography (EEG) than for functional magnetic resonance imaging (fMRI) data (compare EEG to fMRI results, Fig. 3e–g). Given the spatial character of connectivity and fMRI’s immensely superior spatial resolution with respect to EEG’s, this may prima facie seem surprising. This result could in principle stem from a difficulty in handling the dimensionality of fMRI connectivity matrices. However, further analysis clearly points to fMRI’s markedly lower temporal resolution with respect to EEG’s as the true cause - see Fig. 4. This may indicate that the information encoded in brain dynamics can be more important than its spatial aspect in discriminating between physiological and pathological connectivity, and that fMRI’s relatively poor temporal resolution may lead to an over-simplified image of brain connectivity and therefore fail to capture functionally discriminant aspects.

**Fig. 5:**
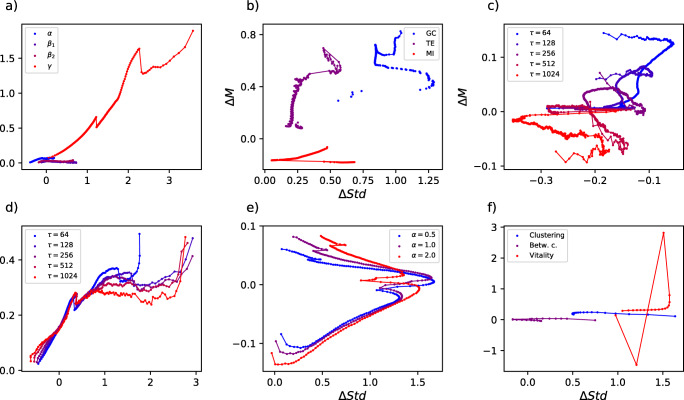
Examples of alternative uses of the proposed methodology. **a**, **b** Analysis of the Schizophrenia (EEG) data set as a function of the frequency bands and the connectivity metrics considered. **c**, **d** Parkinson’s disease (EEG) and Schizophrenia (EEG) data sets, for different values of the length *τ* of the time window used to assess correlations. **e**, **f** Link- and node-based metrics, for the Schizophrenia (EEG) data set

**Fig. 6:**
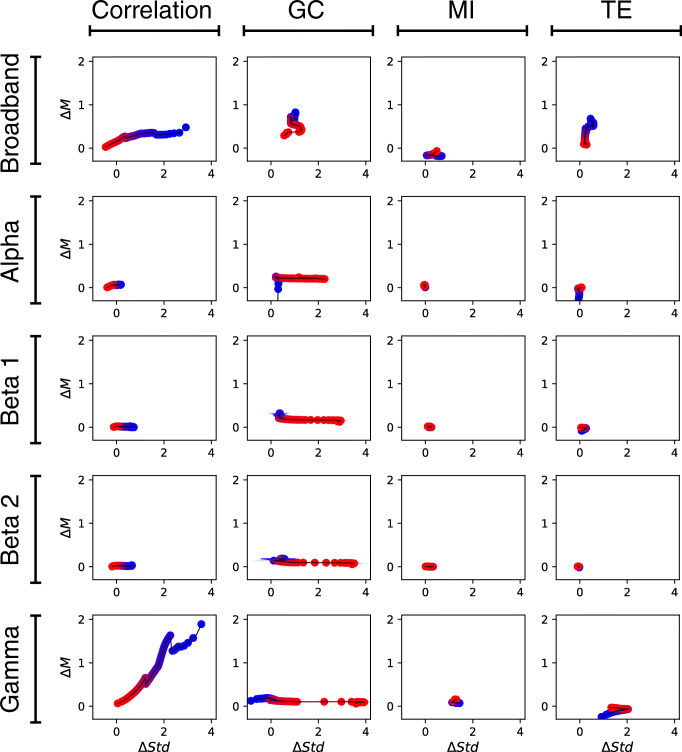
Analysis of the results obtained by the proposed method for the Schizophrenia EEG data set, as a function of the connectivity metric (columns) and of the frequency band (rows). The best differentiation between control subjects and patients, in terms of Δ*M*, is obtained by the linear correlation for high frequencies; on the other hand, larger differences in terms of Δ*Std* are obtained by the Granger Causality

**Fig. 7:**
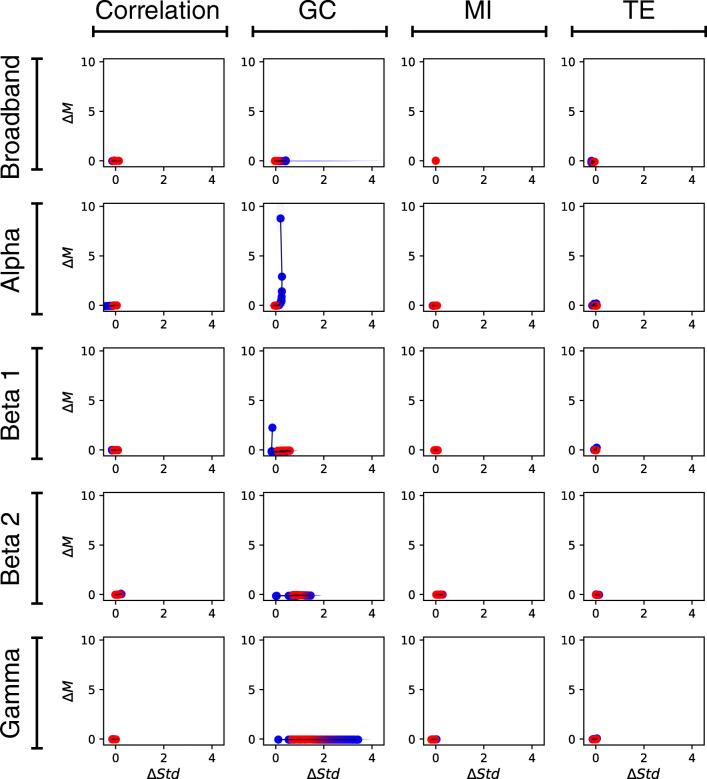
Analysis of the results obtained by the proposed method for the Alzheimer’s Disease EEG data set, as a function of the connectivity metric (columns) and of the frequency band (rows). The best differentiation between control subjects and patients is yielded by the Granger Causality, at lower frequencies in terms of Δ*M*, and at higher frequencies in terms of Δ*Std*

The range of comparisons that the proposed method can help handling far exceeds those between different populations. Notably, the method can be used as a fast *post hoc* validation method in functional network reconstruction. Reconstructing connectivity and network properties from experimental neuroimaging data is a highly non-trivial task, with various discretionary steps for which no accepted theoretical guidelines exist. For instance, there is as yet no principled criterion to define nodes or to choose the most appropriate connectivity metric out of the many available ones, and refining network reconstruction in an iterative manner is a computationally intensive process (Zanin et al. 2012). In a somehow comparable vein, the transform can help understanding what aspects of the recorded signal, e.g. which part of the frequency spectrum of a broad-band signal, contain discriminative information. Figure 5 illustrates some examples of such applications. Panel (a) reports the graphical representation corresponding to filtering the time series at various frequency bands for the Schizophrenia (EEG) data set; panel (b) the use of different connectivity metrics. As previously shown, high frequencies and linear correlations yield the best results (Roach and Mathalon 2008) - see also results for all combinations of frequencies-metrics in Fig. 6. The opposite is observed for Alzheimer’s disease (EEG), see Fig. 7: the alpha band encodes most of the information, as well known in the literature (Moretti et al. 2004). Panels (c) and (d) of Fig. 5 also report the representations for varying lengths *τ* of the time window used to assess the correlation between nodes, respectively for the Parkinson’s disease(EEG) and the Schizophrenia (EEG) data sets. We further show how the methodology can also be applied to high-order network topological metrics, whenever they can be calculated for either links or nodes. In a way similar to link weights, one simply needs to rank these link or node metrics, calculate the between-group Δ*M* and Δ*Std*, and represent the results in a scatter plot. Figure 5e reports the results for the edge betweenness centrality, for the Schizophrenia (EEG) data set, when link weights are raised to an exponent *α*; and panel Fig. 5f results for three node-based metrics, i.e. clustering coefficient, betweenness centrality and vitality.

**Fig. 8:**
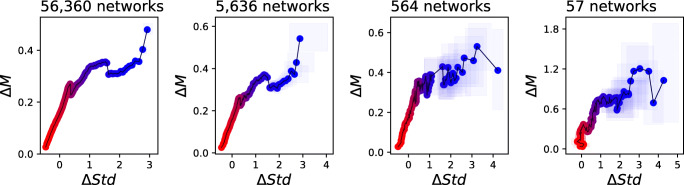
Analysis of the results obtained for the Schizophrenia EEG data set, when a reduced set of networks, drawn at random, are used as input. It can be appreciated that reducing the number of networks does not change the shape of the curve, in spite of an increment in the uncertainty

As a final point, Fig. 8 analyses the sensitivity of the method to the number of available networks. Specifically, the left panel reports the results for all networks extracted from the Schizophrenia EEG data set (i.e., the same as Fig. 3a) , while the three additional panels for subsets of 10*%*, 1*%* and 0.1*%* networks drawn at random. Reducing the number of available instances has a negative effect on the uncertainty of each point, as represented by the larger corresponding boxes; on the other hand, the overall shape of the curve is preserved. The availability of large data sets is thus not a requirement, at least if the analysed structural property has a sufficiently high signal-to-noise ratio; or, in other words, inter-group differences are larger than intra-group ones.

### Application to Other Socio-technical Systems

In order to illustrate the generality of the proposed approach, we here show how it can be applied to two other time-varying socio-technical systems, i.e. international trade and air transport networks.

**Fig. 9:**
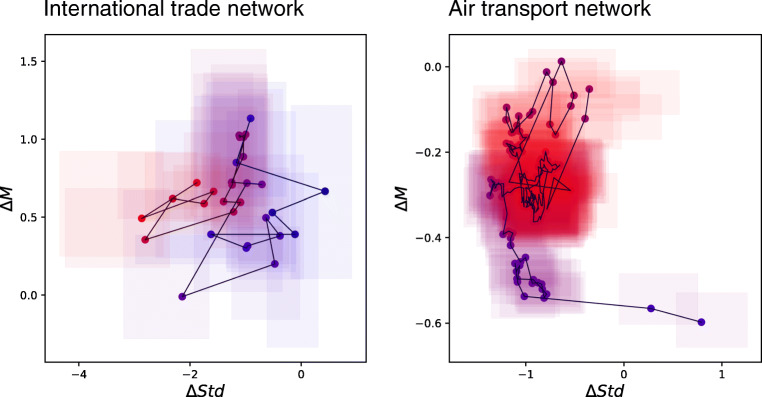
Examples of the application of the proposed methodology to other complex systems

The left panel of Fig. 9 depicts the results for the international trade network between 6 world regions, i.e. Africa, Asia, Australia and New Zealand, Europe, North America, and South and Central America and the Caribbean. Data have been obtained from the World Trade Organisation (WTO) website (timeseries.wto.org), and correspond to the yearly merchandise trade exports for those regions for years 2001 − 2018. The 18 networks have been divided in two groups, respectively corresponding to years 2001 − 2011 and 2012 − 2018. Results indicate that international trade has increased between all regions (0 ≤Δ*M* ≤ 1.2); and that a substantial stabilisation has happened in the strongest connections (Δ*Std* ≈− 3).

The right panel of Fig. 9 corresponds to air transport networks, and specifically on the quarterly number of passengers who traveled between the 20 largest cities in the US. Data have been obtained from the US Department of Transportation (data.transportation.gov/Aviation), and cover years 2009 − 2019. The two groups here correspond to the first and last five years, for a total of 48 networks in each one of them. A generalised reduction in the number of passengers can be appreciated, which especially affects weaker connections (possibly due to spill-over effects to other transportation modes).

## Discussion and Conclusions

In conclusion, we presented a computationally parsimonious graphic method to highlight differences in connectivity between systems under different conditions. This method can be used to capture at a glance essential aspects of the structure and dynamics of biological, technological and economic networked systems, and to iteratively refine their graph representation. From a neuroscientific perspective, we showed that, in most cases, the transform yields an easily identifiable condition-specific geometry of the overall structure of the differences between a given pathology and its corresponding control group in terms of differences in connectivity strength ranking. Our results also highlighted various rather general properties of brain activity and its pathologies, providing important methodological indications: 1) the hierarchy of link strengths in brain connectivity can discriminate between populations, even in the absence of topographic localisation of network links, generally thought to play a prominent role in brain function; 2) some pathologies can be characterised in terms of weak link statistics, and therefore that these links should not be excluded from network analysis as they typically are; 3) information on connectivity dynamics is more important than its spatial definition in discriminating between conditions, indicating that studies using functional magnetic resonance imaging may miss information that may be crucial to the identification of at least certain pathologies; 4) variability has a prominent role in healthy biological systems and in their pathology and should be used to characterise them.

## Information Sharing Statement

The data sets used in this study are freely available and can be downloaded from the corresponding project websites - see the corresponding descriptions in “Data Sets Description”.
